# Acculturation and Naturalization: Insights From Representative and Longitudinal Migration Studies in Germany

**DOI:** 10.3389/fpsyg.2019.01160

**Published:** 2019-05-28

**Authors:** Débora B. Maehler, Martin Weinmann, Katja Hanke

**Affiliations:** ^1^Department Survey Design and Methodology, Leibniz Institute of Social Sciences (GESIS), Mannheim, Germany; ^2^Department of Migration and Mobility, Federal Institute for Population Research, Wiesbaden, Germany

**Keywords:** acculturation, citizenship, immigrant, identity, Germany, naturalization, representative data, longitudinal data

## Abstract

In recent years, Western countries have been experiencing a growing wave of immigration. Due to this development, these countries are facing great challenges in successfully integrating large numbers of immigrants and in preserving social cohesion. Research has already developed several assumptions about and models of how acculturation processes occur. The present contribution aims to investigate the relationship between the acculturation (and acculturation profiles) of immigrants and naturalization in their residence countries. Based on representative and longitudinal data, our investigation is a case study on Germany—one of the main receiving countries in recent years. Results show that acculturation in the country of residence is crucial for immigrants' motivation to take up citizenship. Likewise naturalization leads to an increase in identification with the residence country.

## Introduction

Migration flows have been increasing worldwide in recent years. Western countries, in particular, are confronted with growing numbers of immigrants. For example, permanent migration to member countries of the Organization for Economic Co-operation and Development (OECD) increased from 4.3 million in 2014 to ~4.8 million in 2015 (Organisation for Economic Co-operation Development (OECD), 2016). These developments have led to growing concerns in Western societies that borders will be insecure, that immigrants will burden the social welfare systems, and that some will not integrate. Moreover, an increase in anti-immigrant rhetoric and right-wing populism can be observed in these countries (Alba and Foner, 2015; Organisation for Economic Co-operation Development (OECD), 2016). Thus, Western countries are not only facing great challenges in successfully integrating large numbers of culturally, linguistically, and religiously diverse immigrants into their educational systems, labor markets and citizenry (Guerra et al., 2015), but also in preserving social cohesion.

Hence, politicians are increasingly calling on immigrants to demonstrate their loyalty to the receiving country and to show that they share its values, identify with it, or want to become citizens (e.g., Martinovic and Verkuyten, 2012). Against this background, a number of questions arise. For example, can immigrants develop an attachment to a country of which they are not yet full members (i.e., citizens)? And can naturalization be used as an instrument to support the acculturation of immigrants, or is the motivation to undergo naturalization predicted rather by acculturation processes?

Several social science studies, particularly in the US, have explored the reasons why immigrants undergo naturalization (Chiswick and Miller, 2008). The focus of these studies has mainly been on the role of immigrants' sociodemographic characteristics (e.g., length of residence, language proficiency, and employment status) and on (migration) policies in the country of origin and the receiving country (e.g., Ersanilli and Saharso, 2011; Vink et al., 2013). In addition, numerous psychological studies have invested effort into assessing immigrants' acculturation. However, as Painter (2013) concluded in her literature review, there was an overall lack of suitable data and empirical studies investigating the relationship between naturalization (i.e., the take-up of citizenship of the residence country) and acculturation.

The present study aims to contribute to closing this gap by investigating the link between the naturalization process and acculturation. We focus on Germany as a case study for all our analyses for the following reasons: first, in recent years, the country has become one of the most important migration destinations in the world [Organisation for Economic Co-operation Development (OECD), 2016]; second, it has recently been transformed into a modern immigration country [Expert Council of German Foundations on Integration Migration (SVR), 2015], third, it is facing the challenges of integrating a large number of immigrants who have come to Germany in the past few years; fourth, the facilitation of naturalization is currently the subject of intensive debate, especially with regard to the toleration of dual citizenship (Weinmann, 2016). In Germany, to be eligible for naturalization, most immigrants have to give up their previous citizenship. Among other things, they must also have been resident in Germany for at least 8 years; they must be able to support themselves and their families without recourse to social assistance or long-term unemployment benefit; they must demonstrate that they have an adequate knowledge of German; and, by passing a naturalization test, they must prove that they have a basic knowledge of the German legal and social system and of living conditions in Germany (see section 10 of the German Nationality Act). Using Germany as a case study, we hope to provide helpful insights for other receiving countries facing the challenge of integrating immigrants.

## Acculturation

Acculturation is a broad process of psychological and socio-cultural adaptation following intercultural contact. Berry (1997), for instance, defines acculturation as a process of cultural and psychological changes that occur as a result of interactions between two culturally different groups. This process can lead to changes at different levels (e.g., behavior or cognition) (Chirkov, 2009; Rudmin, 2009). From a psychological perspective, the focus is on the psychological significance and mechanisms of acculturation at the individual level (see also Chirkov, 2009). Thus, Chirkov (2009, p. 94; emphasis in original) defines acculturation as “a process *executed* by an agentic individual (it is not a process that *happens to* an individual) after meeting and entering a cultural community that is different from the cultural community where he or she was originally socialized.”

There are several assumptions about how this acculturation process works. Acculturation models differ, for instance, in terms of their dimensionality and domains. Previous research has mainly used two-dimensional models. According to these models, maintenance of the culture of origin and adaptation to the receiving country is structured orthogonally. As a result, an immigrant can, for example, simultaneously identify with the culture of origin and the culture of a new country. Moreover, the research literature uses a number of different domains to measure individual acculturation. They include, in particular: attitudes to acculturation (such as acculturation orientation; e.g., Berry, 1997); cognitive competencies (the acquisition of knowledge and skills, particularly linguistic skills; e.g., Jasinskaja-Lathi and Liebkind, 2007); social contact (e.g., Berry, 1997); behavioral repertoire (such as food, leisure behavior; e.g., Ryder et al., 2000); structural placement (e.g., in the education system or on the labor market; e.g., Esser, 2006); and identity (or sense of belonging; e.g., Hutnik, 1986).

To meaningfully describe the acculturation process, acculturation profiles (or typologies) are often derived. These profiles can be based on one domain (e.g., identity) or on multiple domains (e.g., identity and language). The approach of John Berry (1997) is the most prominent in this regard. He proposed four acculturation profiles that result from the combination of the dimensions of the culture of origin and the culture of the residence country: *assimilation* (a weak orientation toward the culture of origin and a strong orientation toward the culture of the residence country); *separation* (a strong orientation toward the culture of origin and a weak orientation toward the culture of the residence country); *integration* (a strong orientation toward the culture of origin and the culture of the residence country); and *marginalization* (a weak orientation toward both cultures).

Similarly, Phinney et al. (2001, p. 498) used the typology approach to describe four acculturation profiles of young immigrants in the United States, Israel, Finland, and the Netherlands, namely, “integrated identity, assimilated identity, separated identity, and marginalized identity.” The authors used the term *ethnic identity* to describe immigrants' identification with the culture of origin, and the term *national identity* to describe their identification with the new society (Phinney et al., 2001). More specifically, Phinney and Baldelomar (Phinney and Baldelomar, p. 173) defined ethnic identity as “a sense of peoplehood based on one's ancestry and associated with one's cultural values and traditions,” and *national identity* as “a sense of membership in a sovereign political entity.” For example, for Turkish immigrants residing in Germany, an ethnic identity would mean that they identify with Turkish cultural values and traditions, whereas for native Germans, ethnic identity would mean that they identify with German cultural values and traditions. Turkish immigrants who have a sense of membership in Germany have a German *national identity*. Accordingly, whereas native Germans' ethnic and national identities are the same, these immigrants have a Turkish ethnic identity and a German national identity. Nonetheless, it should be emphasized that—for first-generation immigrants in particular—the formation of a national identity is an advanced step in the process of adaptation to a new culture or country, and that it is not necessarily achieved—or achievable. Unlike other acculturation domains, such as language acquisition or behavioral adaptation that can be controlled by individuals themselves, identification with, or feeling attached to, the receiving country cannot be controlled by individuals, because it is based on deeper psychological processes that unfold over time (Ward, 2006; Maehler, 2012).

So far the relationship between acculturation profiles and naturalization has scarcely been investigated (but see Hou et al., 2015 for Canada). In the following section, we will describe general research findings about the relationship between naturalization and acculturation.

## Naturalization and Acculturation

Several lines of research have suggested that naturalization, and thus citizenship of the country of residence, is a *prerequisite* for successful acculturation, and particularly for integration. Studies show, for instance, that immigrant naturalization is positively correlated to the individual level of integration, for example in terms of educational attainment, occupational status, language skills, inter-ethnic friendship or marriage (e.g., Portes and Curtis, 1987; Liang, 1994; Yang, 1994a), and the intensity of social interactions with natives (e.g., Diehl and Blohm, 2003). By contrast, length of residence has been found to have an ambivalent impact. Whereas, some studies have found positive correlations between the length of residence and naturalization, others have found negative effects for immigrants with a very long duration of stay (e.g., Liang, 1994; Yang, 1994b; Diehl and Blohm, 2003; Hochman, 2011). Against this background, it can be postulated that the process of naturalization supports the acculturation of immigrants in the receiving society. On the other hand, the same lines of research have provided evidence that naturalization is more probable for immigrants who are culturally and economically integrated and is, therefore, an *outcome* of the acculturation process (e.g., Joppke, 1999; Wunderlich, 2005; Hochman, 2011; Vink et al., 2013). Hochman (2011), for instance, found a positive correlation between labor migrants' sense of belonging and their intention to apply for naturalization. However, to our knowledge, the way in which naturalization affects acculturation has not yet been examined in a longitudinal study.

In the present paper we aim to investigate the relationship between acculturation and the motivation to undergo naturalization. More specifically, we will examine whether naturalization can be predicted by acculturation, or whether acculturation is an outcome of naturalization. We will use the domain of identity (see above) as an indicator for acculturation.

## Hypotheses

To verify the relationship between naturalization and acculturation, we will conduct two studies. Study 1 is based on representative data and investigates the relationship between naturalization and acculturation for immigrants in Germany. Study 2 investigates the relationship between naturalization and acculturation over time and explores in an individual approach the relationship between naturalization and acculturation profiles.

In Study 1, we will compare the acculturation of immigrants by naturalization status (non-naturalized, undergoing naturalization, naturalized) based on representative, cross-sectional data on the immigrant population in Germany. We propose the following hypothesis:

*H1*. Immigrants who identify primarily with the country of residence or with both the country of residence and the country of origin are more likely to apply for naturalization (i.e., undergo the naturalization process) or to be already naturalized than immigrants who identify primarily with their country of origin.

Based on longitudinal data collected in Study 2, we will then investigate whether naturalization leads to greater acculturation. Our hypothesis is as follows:

*H2*. Naturalized immigrants show a higher degree of acculturation 1 year after naturalization, whereas the degree of acculturation of non-naturalized immigrants does not change over a 1-year period.

In a further step, based on the data collected in Study 2, we will explore what acculturation profiles are characteristic of naturalized immigrants compared to non-naturalized immigrants, and we will measure changes in their profiles over time. We propose the following hypotheses:

*H3a*. Immigrants who have been naturalized recently are more often represented in acculturation profiles that have higher acculturation scores (i.e., an assimilation or integration profile) than are non-naturalized immigrants.

*H3b*. Naturalized immigrants with acculturation profiles that have lower acculturation scores (i.e., a separation or an indifference profile) show an increase in identification with Germany 1 year after naturalization, whereas identification with Germany on the part of non-naturalized immigrants with such profiles does not change over a 1 year period. For both groups, identification with the culture of origin does not change over a 1-year period.

In this context, we will further explore whether naturalized immigrants with dual citizenship (i.e., citizenship of their country of residence and of their country of origin) differ in terms of acculturation profile from naturalized immigrants who have only German citizenship.

**Figure 1:**
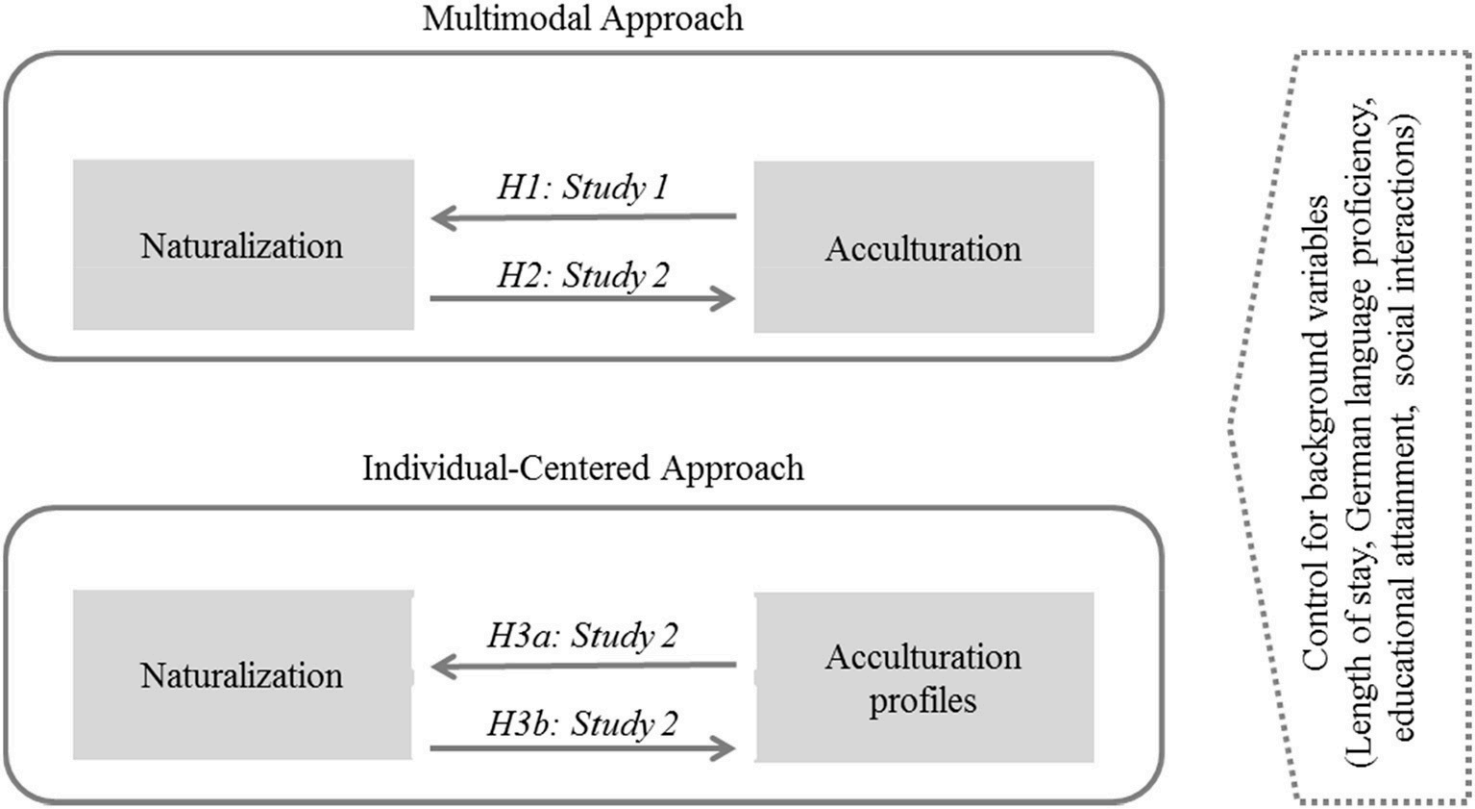
Design to investigate the relationship between naturalization and acculturation.

Figure 1 summarizes the hypotheses to be tested in Study 1 and Study 2.

## Study 1

Our first study served to investigate the relationship between naturalization status and acculturation (identity). Using representative data allowed us to test whether our assumptions are generalizable to the immigrant population in Germany.

### Data and Method

Both Study 1 and Study 2 followed the “Ethical Principles of Psychologists and Code of Conduct” of the American Psychological Association (APA) and were carried out in accordance with the relevant German statutory provisions. All participants gave informed consent in accordance with the Declaration of Helsinki. In the case of the longitudinal study (Study 2), participants also gave their consent to be re-contacted after 1 year. The German Federal Data Protection Commissioner was responsible for overseeing compliance with and application of data protection regulations in Study 1. Participation in both studies was voluntary. The maintenance of confidentiality was assured. Participants had the right to withdraw from the studies at any time without any consequences and were supplied with contact details to request further information. Finally, neither the guidelines of the respective institutions at which Study 1 and Study 2 were conducted nor the national statutory provisions required that surveys of this type should be reviewed and approved by an ethics committee.

In Study 1, we tested our first hypothesis using data from the 2011 BAMF Naturalization Study (Federal Office for Migration Refugees (BAMF), 2012; Weinmann et al., 2012), which was conducted by the German Federal Office for Migration and Refugees (BAMF). The study was a representative, cross-sectional survey of the immigrant population in Germany. It was designed with the aim of comparing non-naturalized and naturalized immigrants as well as immigrants undergoing naturalization. This allowed us to obtain a generalizable picture of the link between immigrants' naturalization status and their acculturation processes.

The BAMF Naturalization Study provides a unique dataset comprising data from 1,133 interviewees of the largest immigrant groups in Germany with different naturalization statuses: non-naturalized immigrants (i.e., persons of foreign nationality, who fulfill the necessary residence requirement for naturalization, *n* = 411), immigrants undergoing the naturalization process (*n* = 403), and naturalized immigrants (*n* = 319). The sample included 47.3% female participants. The average age of the interviewees was 37.25 years (*SD* = 13.20; range: 18–85 years). Participants rated their respective language proficiency as good (*M* = 1.80; *SD* = 0.84; scale: 1–6); 73.9% were born abroad (average length of residence: 21.35 years; *SD* = 11.54; range < 1–61 years; age at immigration: 19.01 years; *SD* = 11.46). The main countries of origin in the sample were Turkey and the former Yugoslavian countries. Regarding educational attainment (coded according to ISCED), a high (43.4%) or low (33.0%) level of education was most common.

The data were collected in 2011 using a mixed-mode method: computer-assisted telephone interviews were conducted with naturalized and non-naturalized persons nationwide and computer-assisted personal interviews were conducted with persons undergoing the naturalization process. A multistage sampling procedure was used to recruit immigrants for the telephone interviews. In the first stage, households were randomly sampled using an onomastic procedure whereby immigrants were identified in telephone directories by their names. In the second stage, target persons were identified through a telephone screening interview. If there were at least two persons from the target group living in the household, the interviewee was selected in a third stage using a computer-based random selection process. For immigrants undergoing the naturalization process, it was possible to generate a random sample based on a register of immigrants who had applied for naturalization but had not yet been naturalized. This register was compiled on the basis of address lists provided by the relevant public authorities responsible for naturalization. The address lists made it possible to conduct computer-assisted personal interviews.

The random sampling for all three target groups was devised disproportionately in order to ensure a sufficient number of interviews with immigrants from the five most important regions of origin of naturalized and non-naturalized immigrants living in Germany (i.e., Turkey; the former Yugoslavian countries; Greece and Italy; Afghanistan, Iran, Iraq; the Russian Federation, Ukraine, Belarus). To ensure a representative analysis, the data were weighted based on official statistics on naturalizations and foreign nationals in Germany (Pupeter et al., 2011). As German language proficiency is a prerequisite for naturalization, the interviews with naturalized immigrants and immigrants undergoing the naturalization process were conducted in German. Non-naturalized immigrants were given the option of being interviewed in German or in another language (Farsi, Greek, Italian, Russian, Serbo-Croatian, or Turkish) because it was expected that not all target persons would have a sufficient command of German (16% of the interviews were conducted in one of these languages). Identity was measured with one question: “With which country do you feel a greater affinity?” The participants could select one of the following response options: (1) primarily with the country of origin, (2) with both countries equally, and (3) primarily with Germany.

### Results

Descriptive analyses revealed that 48.5% of all naturalized respondents, 63.6% of respondents undergoing the naturalization process, and 35.7% of all non-naturalized respondents identified primarily with Germany. Only 4.3% of all naturalized respondents and 1.8% of those undergoing the naturalization process identified primarily with the country of origin, whereas the figure for all non-naturalized respondents was 11.7%. Finally, almost half (47.2%) of the sample of naturalized immigrants and over half (52.6%) of the sample of non-naturalized immigrants identified with both countries equally, whereas only about one third (34.6%) of those undergoing the naturalization process did so.

To test whether immigrants' level of acculturation (operationalized as identification) in the country of residence was correlated to their naturalization status (non-naturalized, undergoing the naturalization process, naturalized) (*H1*), we conducted multinomial logistic regression analyses using naturalization status as a dependent variable (coded as 0 = non-naturalized; 1 = undergoing the naturalization process; 2 = naturalized) and identification as an explanatory variable (using Stata 12). Sociodemographic background characteristics (length of residence, educational attainment, and German language proficiency) and the intensity of social interactions with native Germans were included in the second analysis as control variables. The findings in Table 1 show that respondents' identification with Germany was positively correlated to naturalization status *(H1)*. Hence, respondents who identified primarily with Germany or with both Germany and the country of origin were more likely to be undergoing the naturalization process or to be already naturalized than respondents who identified primarily with their country of origin (Model 1), even when we controlled for covariates, such as language skills or lengh of residence (Model 2). Interestingly, this effect was stronger for respondents undergoing the naturalization process than for respondents who were already naturalized.

**Table 1:** Multinomial logistic regression models predicting naturalization status by identity, controlling for background variables (naturalization status: 0 = non-naturalized; 1 = undergoing naturalization process; 2 = naturalized).

	**Model 1**				**Model 2**			
	**Undergoing naturalization process**		**Naturalized**		**Undergoing naturalization process**		**Naturalized**	
**Predictors**	**OR**	***SE***	**OR**	***SE***	**OR**	***SE***	**OR**	***SE***
Identity (Ref.: Primarily with country of origin)								
With both countries equally	4.36^**^	2.36	2.42^*^	1.09	8.94^***^	5.40	3.80^**^	1.73
Primarily with Germany	11.78^***^	6.33	3.66^**^	1.66	22.75^***^	13.82	5.91^***^	2.75
Length of stay					0.90^***^	0.01	0.95^***^	0.01
German language proficiency					0.39^***^	0.06	0.40^***^	0.07
Educational attainment (Ref.: low)								
Medium					0.50^*^	0.15	1.48	0.49
High					0.38^***^	0.11	1.73	0.53
Social interactions with native Germans					0.79^*^	0.08	0.83^*^	0.08
Constant	0.15^***^	0.08	0.29^**^	0.12	33.81^***^	32.13	6.94^*^	5.34
Pseudo *R*^2^		0.034				0.160		
Prob > *F*		0.000				0.000		
*N*		1,122				1,085		

*OR, odds ratio. Length of stay: years; if born in Germany: age in years. German language proficiency: index constructed from respondents' self-reported speaking, reading, and writing proficiency, ranging from 1: very good to 6: non-existent (mean). Educational attainment: ISCED. Social interactions with native Germans: index constructed from respondents' self-reported contact intensity in the domains of family and relatives, friends and acquaintances, and neighbors ranging from 1: never to 6: daily*.

* p ≤ 0.05;

** p ≤ 0.01;

*** *p ≤ 0.001*.

*Source: 2011 BAMF Naturalization Study (Weinmann et al., 2012)*.

## Study 2

Study 2 investigated the relationship between naturalization and acculturation over time using path models, as well as exploring the relationship between naturalization and acculturation profiles over time using an individual-centered approach.

### Data and Method

The longitudinal sample (*N* = 505) comprised two sub-samples: the first sub-sample consisted of persons who were recently naturalized (*n* = 279); the second sub-sample was a control group (*n* = 226) comprising non-naturalized persons. The first wave was conducted in 2007. Whereas, recently naturalized citizens were recruited in public authorities and at naturalization ceremonies, non-naturalized immigrants were recruited in public institutions (e.g., the civil service, associations) or at public events. The average amount of time that passed between the naturalization of the respondents and the survey was 48 days. A mixed-mode method of data collection was used (telephone interview: 77.8%; self-administered paper-and-pencil questionnaire: 22.2%). The survey was conducted in German.

In the second wave in 2008, 161 naturalized and 90 non-naturalized immigrants remained in the sample (response rate: 49.7%). Regarding possible attrition effects, we tested for background variables, such as gender, age, length of residence, education, and German language proficiency. The analyses showed one effect, namely for education: the probability of dropping out of the second wave was higher for persons with a low level of education [χ^2^_(2, N = 505)_ = 18.99, *p* < 0.001]. Furthermore, a re-test effect could not be identified (*n.s*.).

For the analyses based on the individual-centered approach (see Hypotheses 3a and 3b), the sub-samples were parallelized (Field and Hole, 2006) with regard to length of residence, age, educational attainment, place of birth, and method of data collection. In this way, changes could be attributed to the independent variables and not to structural differences between samples. Thus, for the first time of measurement, 241 naturalized and 138 non-naturalized immigrants were retained in the respective sub-samples. As reported above, for the second time of measurement (longitudinal analyses) 251 participants were retained in the sample. To test Hypotheses 2 and 3 (a and b), we nevertheless used the parallelized dataset (141 naturalized and 80 non-naturalized immigrants), as this procedure excluded only 8% of the total sample and the use of different datasets could be avoided.

The longitudinal sample included 60.2% female participants; the average age of the participants was 32.18 years (*SD* = 10.18; range: 17–66 years). The participants' self-reported German language proficiency was good (*M* = 1.77; *SD* = 0.72; scale: 1 [*very good*] to 6 [*fail*]); 86% were born abroad (average length of residence: 12.9 years; *SD* = 7.93; range < 2–38 years; age at immigration: 20.58 years; *SD* = 9.88). The main countries of origin were Turkey, the Russian Federation, and Poland. With regard to educational attainment (coded according to the International Standard Classification of Education, ISCED), high (53.9%) and medium (45.2%) levels were most common.

Acculturation was assessed on the basis of the domain of identity. Identification with the culture of origin (α = 0.88) and with Germany was operationalized using a six-item scale (α = 0.87). The scale included items such as: “I feel German,” and “I feel a strong affinity with German culture.” The scale ranged from 1 (*strongly disagree*) to 5 (*strongly agree*). Higher values indicate stronger identification. The invariance of the scales has been reported in Maehler and Schmidt-Denter (2013). For the further analyses, sociodemographic background characteristics (e.g., gender, age, language proficiency, educational attainment, country of origin, length of residence) were also assessed. A short overview of both studies is available in the supplement (Table A1).

### Results

#### One Year After Naturalization: Does Naturalization Predict Acculturation?

To test the hypothesis (*H2*) that naturalization predicts acculturation, an indirect effects path model was tested with manifest variables using R Studio with the lavaan package (Rossel, 2012). We used the observed mean of identification with Germany (i.e., national identity) soon after naturalization as the predictor in Model 1. In Model 2, the predictor was the observed mean of identification with the culture of origin soon after naturalization. The outcome variable in both models was the observed mean of both scales 1 year after naturalization. The following analyses are based on 1,000 bootstraps iterations using a bias corrected and accelerated confidence interval (BCa 95% CI).

**Figure 2:**
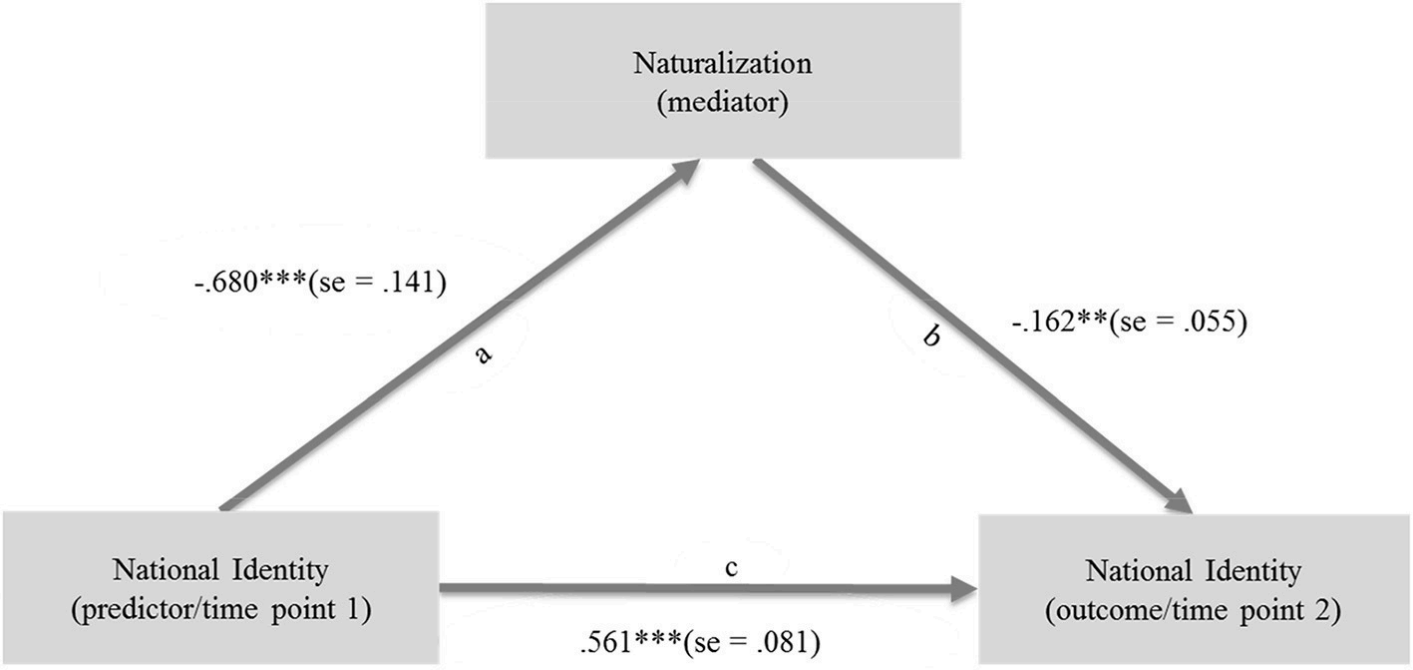
National identity 1 predicted National Identity 2 via Naturalization (categorical: naturalized [0] vs. non-naturalized [1]), unstandardized regressions weights. Indirect effect: 0.110; se = 0.046, z = 2.379; *p* = < 0.05; 95% confidence interval: [0.041, 0.218]. ^***^p < 0.001, ^**^p < 0.01.

Figure 2 shows the results for the model 1: the degree of national identification at the second time of measurement was directly and significantly affected by the degree of national identification at the first time of measurement (path c, direct effect *B* = 0.561, *SE* = 0.081, z = 6.963, *p* = < 0.001). Naturalization positively influenced the degree of national identification 1 year later (direct effect *B* = −0.162, *SE* = 0.055, z = −2.965, *p* = < 0.05).

Furthermore, in accordance with the assumption (*H1*) tested in Study 1, the degree of national identification at time point 1 also had a positive effect on naturalization (path a, direct effect *B* = 0.680, *SE* = 0.141, z = −4.829, *p* = < 0.001). The model explained ~55% of the variance in national identification 1 year after naturalization. Naturalization accounted for 30% of the variance. Following Cohen (1988) this is equivalent to a strong effect. The indirect effect (path a ^*^ b) was *B* = 0.110, *SE* = 0.046, z = 2.379, *p* < 0.05). Even under bias-corrected bootstrapped confidence intervals, the indirect effect remained significant at the 5% level [0.041, 0.218]. We controlled for sex, length of residence, language proficiency, educational attainment, and social interactions with native Germans. Even after controlling for these variables, effects remained stable for Model 1.

As expected, for Model 2, the indirect effect from national identification at time point 1 to national indentification at time point 2 via naturalization was not significant (*B* = 0.002, *SE* = 0.021, z = 0.087, *p* < 0.931). The direct path c was significant: identification with the culture of origin at time point 1 had an impact on identification with the culture of origin at time point 2 (*B* = 0.58, *SE* = 0.064, *z* = 9.029, *p* < 0.001). The direct path a from identification with the culture of origin at time point 1 to naturalization was significant (*B* = 0.357, *SE* = 0.121, z = 2.958, *p* < 0.01). The direct path b from naturalization to identification with the culture of origin at time point 2 remained insignificant (*B* = 0.005, *SE* = 0.054, z = 0.095, *p* < 0.925).

#### Determination and Description of Acculturation Profiles

To derive acculturation profiles for naturalized and non-naturalized respondents, we used a hierarchical cluster analysis following Ward's method (squared Euclidean distance; Ward, 1963). The purpose of cluster analysis is to group individuals so that they are more similar within a group than between groups. In the process, clusters are merged that result in the minimum increase in within-cluster variance. The number of clusters is usually determined taking into account the greatest increase in heterogeneity in a dendrogram. In the present case, this suggested a five-cluster solution. The subsequently conducted discriminant analysis confirmed the *ad-hoc* results of the deterministic cluster analysis. Using discriminant function analysis, a predictive model for group membership was built. As the results show, the five groups detected by the cluster analyses differed significantly with regard to the Identification with Germany scale [*F*_(4, 355)_ = 170.73, *p* < 0.001, η^2^ = 0.34] and the Identification with the Culture of Origin scale [*F*_(4, 355)_ = 223.14, *p* < 0.001, η^2^ = 0.28].

Unlike previous findings, five acculturation profiles (also known in acculturation research as acculturation orientations or strategies) could be differentiated from the relationship between the identity scales. We defined as “separated” those respondents (*n* = 127) who had a strong identification with their culture of origin (*M* = 4.23, *SD* = 0.49) and a less strong identification with Germany (*M* = 3.07, *SD* = 0.57). Two integration profiles were also found: the first, “partial integration,” was represented by respondents who had an average identification with both cultures (*n* = 91; identification with culture of origin: *M* = 3.84; *SD* = 0.49; identification with Germany: *M* = 3.56, *SD* = 0.37); the second, “high integration,” was represented by respondents who had a strong identification with both cultures (*n* = 34; identification with culture of origin: *M* = 4.74; *SD* = 0.22; identification with Germany: *M* = 4.65; *SD* = 0.32). Respondents who identified neither with their culture of origin (*M* = 2.92; *SD* = 0.37) nor with German culture (*M* = 2.94; *SD* = 0.49) were assigned an “indifference” profile (*n* = 73). In the literature, this is also referred to as a “marginalization profile” (e.g., Berry et al., 2006; Rudmin, 2009). The fifth profile that we identified was “assimilation” (*n* = 35), which was characteristic of respondents with a strong identification with German culture (*M* = 4.51; *SD* = 0.49) and a weak identification with the culture of origin (*M* = 2.32; *SD* = 0.67).

Overall, the respondents assigned to the five acculturation profiles did not differ in terms of the sociodemographic background characteristics gender [χ^2^_(4, N = 360)_ = 1.86, *ns*], age [*F*_(4, 344)_ = 1.26, *ns*], educational attainment [*F*_(4, 353)_ = 0.72, *ns*], socioeconomic status [*F*_(4, 186)_ = 1.08, *ns*], place of birth [χ^2^_(4, N = 353)_ = 8.96, *ns*], and culture of origin [χ^2^_(12, N = 311)_ = 20.58, *ns*]. However, they did differ in terms of length of residence [*F*_(4, 324)_ = 6.87, *p* < 0.001, *r* = 0.29], German language proficiency [*F*_(4, 354)_ = 2.54, *p* < 0.05, *r* = 0.17], and language proficiency in the heritage language [*F*_(4, 354)_ = 7.87, *p* < 0.001, *r* = 0.30]. *Post-hoc* tests according to Gabriel (see Field and Hole, 2006) showed that, compared to partially integrated respondents (*M* = 0.49, *SD* = 0.30) and strongly integrated respondents (*M* = 0.40, *SD* = 0.27), respondents with an indifference profile (*M* = 0.68, *SD* = 0.29) had spent a larger proportion of their lives in Germany (*p* < 0.001 for all tests). In addition, indifferent respondents (*M* = 1.70, *SD* = 0.10) were more proficient in German than partially integrated respondents (*M* = 2.22, *SD* = 0.15; *p* < 0.5). With regard to the language of origin, *post-hoc* tests according to Games Howell, which were conducted because no equality of population variance could be assumed, revealed that indifferent respondents (*M* = 2.31, *SD* = 0.14) differed from separated respondents (*M* = 1.48, *SD* = 0.11) and partially integrated respondents (*M* = 1.28, *SD* = 0.20) (*p* < 0.001 for all tests), and that partially integrated respondents differed from respondents with high integration profiles (*M* = 1.91, *SD* = 0.12) (*p* < 0.05).

#### Do Immigrants With Different Acculturation Profiles Differ in Their Motivation for Naturalization?

**Figure 3:**
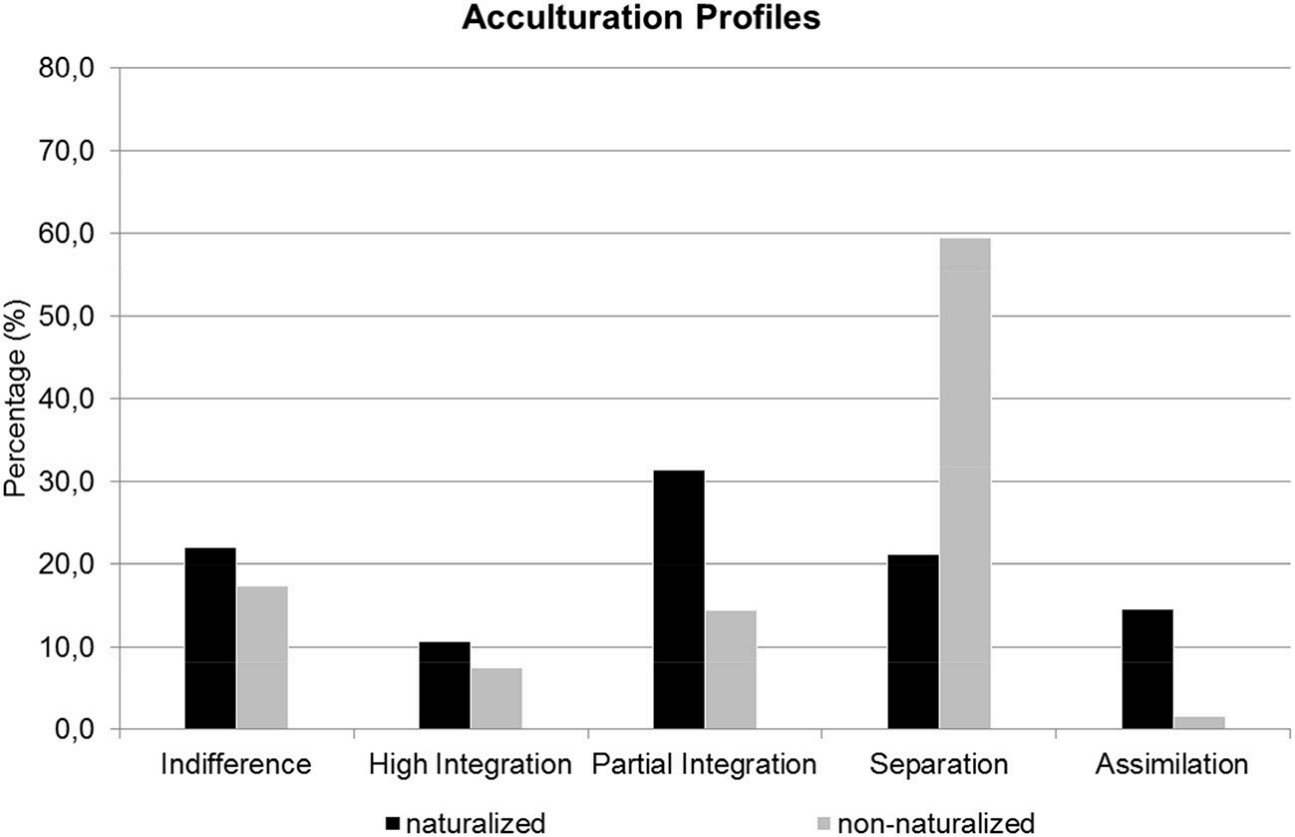
Acculturation profiles of immigrants in Germany.

As proposed in our third hypothesis *(H3a)*, the comparison between naturalized and non-naturalized respondents revealed that naturalized respondents identified more strongly with Germany (see Figure 3): an assimilation or integration acculturation profile was characteristic of these respondents, whereas, once again, a separation profile was characteristic of non-naturalized respondents. A chi-square test showed that disproportionately more naturalized respondents and less non-naturalized respondents were assigned to the assimilation or high integration profile, and disproportionately more non-naturalized respondents and less naturalized respondents were assigned to the separation profile [χ^2^_(4, N = 360)_ = 61.27, *p* < 0.001]. Furthermore, our exploration of the correlation of dual citizenship and acculturation by means of a *t*-test [*t*_(268)_ = 0.25, *p* = 0.799] revealed that the naturalized sub-sample with dual citizenship (*M* = 3.73, *SD* = 0.74) did not differ significantly in their identification with Germany from naturalized respondents who held only German citizenship (*M* = 3.71, *SD* = 0.75).

As expected, the results show that immigrants with an assimilation or integration profile have a higher motivation to undergo naturalization, and immigrants with a separation profile have a lower motivation.

#### One Year After Naturalization: In Which Acculturation Profiles Did Identification Increase?

**Figure 4:**
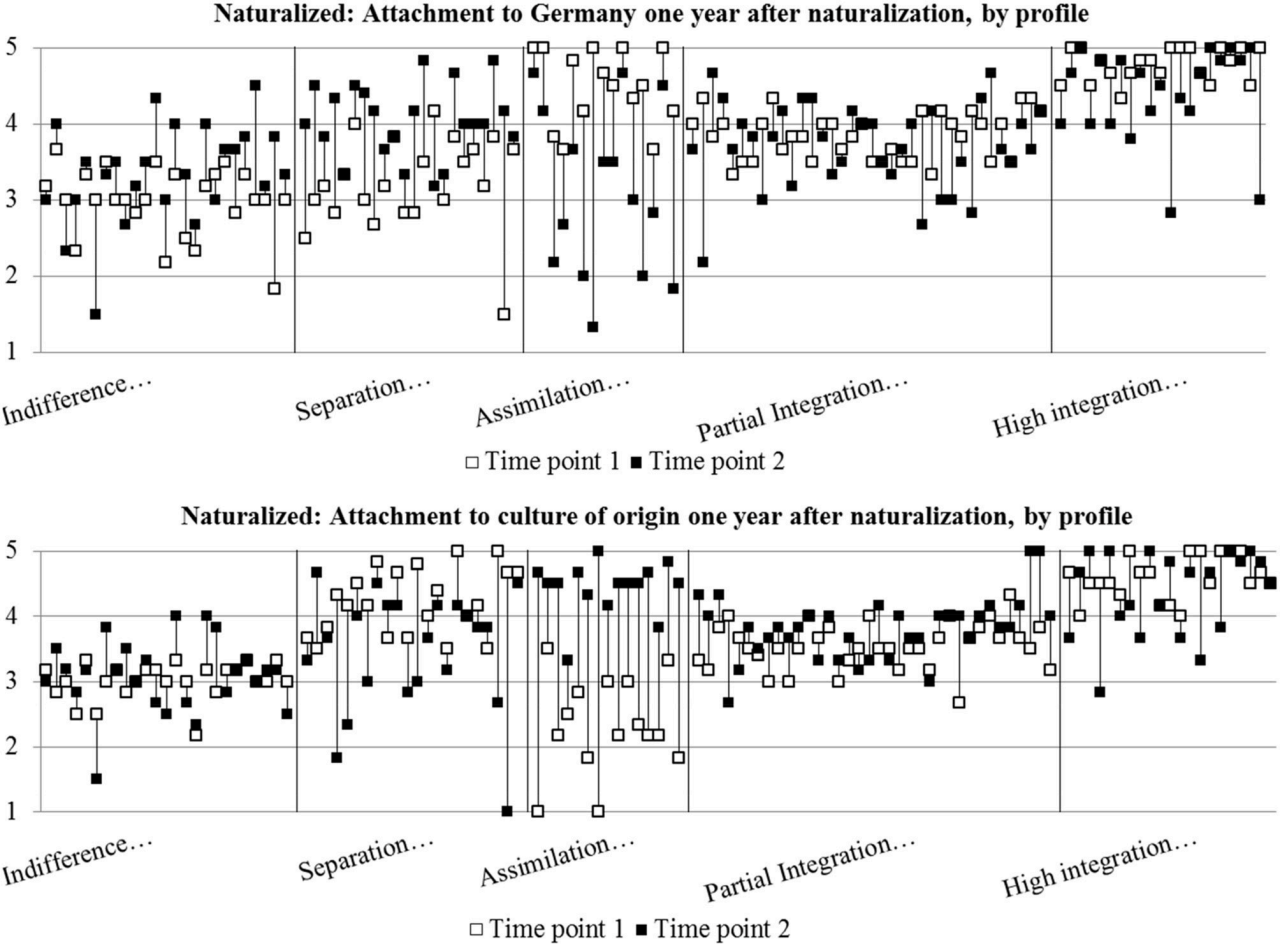
Acculturation profiles of immigrants in Germany 1 year after naturalization.

To better understand the assumptions and the aforementioned results at an individual level, we looked for changes in the profiles over time (*H3b*). Figure 4 shows the values for all naturalized participants at the two points in time ordered by profile. Using a paired sample Wilcoxon sign-rank test (non-parametric test), the results for *naturalized respondents* showed hardly significant changes in the average value for identification with Germany over this period: indifference profile (*n* = 25; T = 85.50, *ns*); partial integration profile (*n* = 36; T = 230.00, *ns*); assimilation profile (*n* = 15; T = 35.00, *ns*); separation profile (*n* = 22; T = 52.00, *ns*). This was the case even when the trends were in the expected directions—for instance, when respondents with a separation profile tended to identify more with Germany after naturalization. Unexpectedly, respondents with a high integration profile identified significantly less with Germany 1 year after naturalization (*n* = 21; T = 13.50, *p* < 0.05, *r* = −0.60). Regarding identification with the culture of origin, no changes were observed in naturalized respondents with a partial integration profile (T = 146.50, *ns*), a high integration profile (T = 47.50, *ns*), or a separation profile (T = 487.00, *ns*). However, respondents with an indifference profile (T = 46.50, *p* < 0.05, *r* = 0.58) or an assimilation profile (T = 2.00, *p* < 0.05, *r* = 0.79) identified more strongly with the culture of origin 1 year after naturalization.

Because of insufficient sample sizes for *non-naturalized immigrants* in the follow-up survey, some acculturation profiles could not be considered in the calculations comparing means from time points 1 and 2 on group level. There were no non-naturalized respondents with an assimilation profile (*n* = 0), and there were so few non-naturalized respondents with a high integration profile (*n* = 3) that they could not be taken into account. For the other profiles, the analyses of identification with Germany showed no significant changes among indifferent respondents (*n* = 7; T = 13.00, *ns*) and separated respondents (*n* = 25; T = 128.00, *ns*). However, there was a significant decrease in identification among partially integrated respondents (*n* = 11; T = 8.00, *p* < 0.05, *r* = −0.70). Regarding identification with the culture of origin, the analyses of the survey data collected after 1 year showed no significant changes among respondents with an indifference profile (T = 9.00, *ns*), a partial integration profile (T = 12.00, *ns*), or a separation profile (T = 88.50, *ns*). These results do not confirm the expectation that naturalized immigrants with acculturation profiles that had lower acculturation scores, such as a separation or an indifferent profile would show an increase in identification toward Germany 1 year after naturalization.

## Discussion

The main purpose of this contribution was to analyze the relationship between acculturation and naturalization of immigrants in the country of residence. We used Germany as an example of a Western country that has experienced strong immigrant inflows in recent years. Based on representative survey data for the largest immigrant groups in Germany, we were able to demonstrate that the degree of acculturation (operationalized as identification) was positively correlated to the decision to apply for naturalization. In other words, respondents who were already naturalized and respondents who aimed to become naturalized citizens identify more strongly with Germany than non-naturalized respondents. This is in line with the referenced literature (see above) based on structural indicators (e.g., job status), which indicates that the decision to apply for naturalization is the culmination of the integration process in Germany. Using longitudinal data, we investigated the relationship between acculturation and naturalization over time. According to our expectations, naturalization leads to an increase in identification with the residence country 1 year after respondents had been naturalized.

Interestingly, our exploratory research also revealed that tolerating dual citizenship would not jeopardize immigrants' identification with their country of residence. Naturalized respondents with dual citizenship did not differ in their degree of identification with Germany compared to naturalized respondents who had only German citizenship. These findings could contribute to the long-standing public debate in Germany on dual citizenship and may have an impact on future political decisions.

However, why do some immigrants undergo naturalization and others do not? We looked for the relationship between acculturation profiles and naturalization, which has not been done in the acculturation literature yet. This allows us to look more deeply into this relationship. Our results, based on an individual-centered approach, revealed that naturalized immigrants were characterized by an assimilation or integration acculturation profile, whereas non-naturalized immigrants tended to have a separation profile. In the context of Germany, it is particularly relevant to see that even people who maintain both orientations (Germany and culture of origin) are motivated to become naturalized or that this concept is feasible. One year after naturalization, we expected changes in identification to have occurred, particularly among respondents with extreme acculturation profiles. For example, we expected that, after naturalization, respondents with a separation profile would identify more strongly with Germany, whereas respondents with an assimilation profile would remain on a similar level of identification. These expectations were not confirmed. Naturalized individuals with an assimilation profile or an indifference profile were even found to identify a little more strongly with the culture of origin.

Regarding the finding that immigrants with a high integration profile identify less with Germany 1 year after naturalization, a reason might be that this decrease might be caused by a ceiling effect. In other words, the level of identification may have already been close to or at the maximum at the time of naturalization, so that no further increase was possible. This interpretation is in line with previously mentioned findings from our cross-sectional study: a strong identification with the country of residence may result in the decision to apply for naturalization. After naturalization, the enthusiasm that led to the decision to apply for citizenship decreases and national identification levels off. In other words: enthusiasm for the country of residence prompts the decision to undergo the naturalization process, but the level of identification normalizes after naturalization. Moreover, when conducting research on the relationship between acculturation and naturalization, it must be kept in mind that eligibility for naturalization is subject to a comparatively large number of preconditions, and that in the case of Germany, for example, applicants must prove that they have achieved a certain degree of integration (e.g., language proficiency and a basic knowledge of the German legal and social system).

Additionally, the perception of the environment can influence the identification with the residence country and therefore the acculturation process. The following remark of a participant in the longitudinal study (Study 2) provides an enlightening insight into the relationship between naturalization and acculturation in the German context:

If I am not naturalized, then I can handle both identities well. Since I speak good German and am integrated, I am frequently asked why I am not naturalized. This is in turn good for my self-esteem and signals that I am welcome here! On the other hand, if I were to become a citizen, many people would ask where I came from and this would in turn put my German identity in question!

At present, more knowledge about adult immigrants' identity development processes could be useful to understand cohesion processes in Western societies. Overall, even though we used a longitudinal approach with a time interval of 1 year, the resulting trends of the different acculturation profiles may not completely generalize over a more prolonged time interval. Some expectations (see above) may be confirmed using a more extensive time period. However, there is also the possibility that they deviate from the present results.

### Limitations and Implications for Future Research

In a study involving individuals with different cultural backgrounds, biases at different levels can occur. It can be assumed that the same factors were measured across all the cultures included in our two studies. However, despite the strength of these studies, there are also some limitations. The recruitment of samples remains a major challenge in migration research. For instance, it is very resource consuming to recruit a representative sample of recently naturalized immigrants. In addition to the often lacking language skills, this is certainly one of the reasons why research in this area is developing slowly.

Moreover, the use of categories to measure identification (e.g., primarily with the country of origin; with both countries equally; primarily with Germany) in the representative study (Study 1), for example, may have been problematic. Future research should use continuous variables (e.g., separate scales for assessing identification with the country of residence and the country of origin).

Furthermore, caution is required when using certain terms. In the relevant literature on the identity of immigrants, for example, the term *ethnic group* (e.g., Phinney, 1989; Phinney and Ong, 2007) in questions dealing with identification with the culture of origin (e.g., in questionnaire items, such as “I have a strong sense of belonging to my own ethnic group”; Phinney and Ong, 2007, p. 276). However, in Germany, *ethnisch* implies exclusion and cannot be used in the same way in German-language surveys as in English-language surveys. Therefore, in our longitudinal study (Study 2), the term “culture of origin” was deliberately chosen instead of “ethnic group.” And when the question dealt with identification with the autochthonous majority society, we used the term *German culture*. By contrast, the representative study (Study 1) used the terms *country of origin* and *Germany*. So far, there is no standardized concept—either in psychology or in other social sciences—for the German context. Nor is there any evidence of the construct validity of other concepts either.

Even though the findings of our studies may be equally relevant for other migrant- receiving countries, our data do not allow us to generalize our results beyond the German immigrant population. It would be interesting for future research to test the impact of national-level political indicators on individual-level acculturation outcomes in a multilevel model for a broad range of countries. Germany has relatively low naturalization rates and a comparatively restrictive naturalization policy in comparison to other OECD countries (e.g., MIPEX, 2010; Organisation for Economic Co-operation Development (OECD), 2011; Sartori, 2011). However, for ecological fallacy reasons, individual-level conclusions cannot be drawn from aggregate-level studies (Robinson, 1950). Nevertheless, several comparative multilevel analyses on naturalization rates and naturalization policies point to the fact that different (i.e., more restrictive or less restrictive) citizenship policies may also have an impact on naturalization (e.g., Dronkers and Vink, 2012; Vink et al., 2013). Furthermore, Ersanilli and Koopmans (2010, p. 785) found in their study on the socio-cultural integration of naturalized and non-naturalized immigrants in the Netherlands, France, and Germany that identification with the country of residence was higher “in countries with accessible citizenship regimes.”

Overall, the results of our longitudinal study (Study 2) provide a very important insight into the relationship between acculturation profiles and naturalization, namely, that acculturation profiles may not be conditional upon naturalization and that emotional stances (in the present studies: identification) can vary over time. However, the extent to which temporal effects influence such measurements did not become clear. Further measurement controlling for fluctuating emotions could be valuable for future research.

Finally, our crucial contribution based on representative findings for immigrants in Germany, show that immigrants who identified with the residence country are more likely to undergo naturalization than immigrants who identified with their country of origin. Moreover, the unique longitudinal data show that the *new* citizenship is likely to be a path to identification with the residence country. In this sense, as questioning in the introduction, naturalization can be used as an instrument to support the acculturation of immigrants.

## Ethics Statement

The authors confirm that both Study 1 and Study 2 followed the “Ethical Principles of Psychologists and Code of Conduct” of the American Psychological Association (APA) and were carried out in accordance with the relevant German statutory provisions. All participants gave written informed consent in accordance with the Declaration of Helsinki. In the case of the longitudinal study (Study 2), all participants also gave their written consent to be re-contacted after 1 year. The German Federal Data Protection Commissioner was responsible for overseeing compliance with and application of data protection regulations in Study 1. Participation in both studies was voluntary. The maintenance of confidentiality was assured. Participants had the right to withdraw from the studies at any time without any consequences and were supplied with contact details to request further information. Finally, neither the guidelines of the respective institutions at which Study 1 (University of Cologne) and Study 2 were conducted (Study 1: 2007–2008; Study 2: 2011) nor the German national statutory provisions required that surveys of this type should be reviewed and approved by an ethics committee. Therefore there was no institutionalized ethical committee at the time of studies were carried out at the corresponding university and federal office available (and consequently cannot be named here). To date, there is generally no standard procedure for ethical approval required for survey research in Germany, but Human Ethics Committees are in the phase of being developed. There are guidelines however (Safeguarding Good Scientific Practice) stated by the German Research Foundation to which we and every researcher adheres to. These guidelines also include ethical principles of research.

## Author Contributions

All authors are experts in migration and integration research. DM had the lead for the manuscript. DM was the project manager of the longitudinal study (designed the study and collected the data). MW was involved in the design and data collection of the representative study. DM wrote the theoretical background. DM and MW performed the analyses, wrote the results and the discussion. DM, MW, and KH revised the manuscript. All authors read and approved the final manuscript.

### Conflict of Interest Statement

The authors declare that the research was conducted in the absence of any commercial or financial relationships that could be construed as a potential conflict of interest.
