# Supplementary material for: Acculturation and Naturalization: Insights From Representative and Longitudinal Migration Studies in Germany
Source: Front Psychol. 2019 May 28;10:1160. doi: 10.3389/fpsyg.2019.01160 (PMC6546881; doi:10.3389/fpsyg.2019.01160)
Supplement: Supplementary file 1 [file Table_1.docx]

Supplement/Appendix

**TABLE A.1** **|** Overview of data collection and sample characteristics of both studies.

|  | **Study 1** | **Study 2** |
| --- | --- | --- |
| **Data collection** |  |  |
| Implemented by | Federal Office for Migration and Refugees (BAMF)/Author and others | Author/University of Cologne |
| Design | Cross-sectional; representative for most relevant migrant groups in Germany | Longitudinal |
| Survey year | 2011 | Wave 1: 2007; Wave 2: 2008 |
| *N* | 1,133 (319 naturalized; 403 undergoing the naturalization process; 411 non-naturalized) | Wave 1: 360; Wave 2: 221 (141 naturalized; 80 non-naturalized) |
| Method of data collection | Mixed mode: computer-assisted telephone interview (naturalized and non-naturalized) and computer-assisted personal interview (undergoing the naturalization process); survey languages: German (all three target groups), Farsi, Greek, Italian, Russian, Serbo-Croatian, Turkish (only non-naturalized; 16%) | Mixed mode (telephone interview: 77.8%; paper-and-pencil questionnaire: 22.2%); Survey language: German |
| Course of investigation | Response rates: telephone interviews 33% (of all contact interviews; afterwards, screening for relevant groups), personal interviews 31% | Response rate > 60%; no re-test effect |
| Operationalization of identity | Identity measured with one item: ‘With which country do you feel a greater affinity?’ (primarily country of origin/ both countries equally/primarily Germany) | Ethnic and national identity measured with 6-item scale (α = .88; α = .87). Examples of items: “I feel German.” “I feel strong attachment towards the German culture.’ ” Scale ranged from 1 (*strongly disagree*) to 5 (*strongly agree*). |
| **Sample** |  |  |
| Female (share) | 47.3% | 60.2% |
| Age (average) | 37.25 years (*SD* = 13.20; range: 18–85 years) | 32.18 years (*SD* = 10.18; range: 17–66 years) |
| Language proficiency (self-reported) | 1.80 (*SD* = 0.84; scale 1 to 6) | 1.77 (*SD* = 0.72; scale: 1 to 6) |
| Educational attainment (ISCED) | 33.0% (low), 23.6% (medium), 43.4% (high) | 0.5% (low), 45.2% (medium), 53.9% (high) |
| Country of origin (main groups) | Turkey, former Yugoslavian countries | Turkey, Russian Federation, Poland |
| Place of birth | Abroad: 73.9% | Abroad: 86% |
| Age at immigration  (years) | 19.01 years (*SD* = 11.46) | 20.58 years (*SD* = 9.88) |

*Notes*. A detailed description of Study 1 is provided by Weinmann, Becher and Babka von Gostomski (2012), and of Study 2 by Author (2012). Study 2: Each participant was interviewed with the same data collection mode in the first and second waves. Assumptions of construct equivalence are supported and provided in the technical reports by Pupeter, Stadler, and Schneekloth (2011) for Study 1 and by Maehler (2012) for Study 2. For the sample descriptions and the mode of data collection of Study 2, see the longitudinal sample in Wave 2. In order to assess their German language proficiency, participants in both studies were asked to specify how well (1 = *very good* to 6 = *unsatisfactory*) they could speak, read, and write German (mean).
